# Fermionic quantum processing with programmable neutral atom arrays

**DOI:** 10.1073/pnas.2304294120

**Published:** 2023-08-22

**Authors:** D. González-Cuadra, D. Bluvstein, M. Kalinowski, R. Kaubruegger, N. Maskara, P. Naldesi, T. V. Zache, A. M. Kaufman, M. D. Lukin, H. Pichler, B. Vermersch, Jun Ye, P. Zoller

**Affiliations:** ^a^Institute for Theoretical Physics, University of Innsbruck, 6020 Innsbruck, Austria; ^b^Institute for Quantum Optics and Quantum Information of the Austrian Academy of Sciences, 6020 Innsbruck, Austria; ^c^Department of Physics, Harvard University, Cambridge, MA 02138; ^d^Department of Physics, University of Colorado, Boulder, CO 80309; ^e^Joint Institute for Laboratory Astrophysics, University of Colorado and National Institute of Standards and Technology, Boulder, CO 80309; ^f^Université Grenoble Alpes, CNRS, Laboratoire de Physique et Modélisation des Milieux Condensés, Grenoble 38000, France

**Keywords:** fermionic quantum processor, digital quantum simulation, tweezer arrays, quantum chemistry, lattice gauge theories

## Abstract

Simulating the properties of many-body fermionic systems is an outstanding computational challenge relevant to material science, quantum chemistry, and particle physics.-5.4pc]Please note that the spelling of the following author names in the manuscript differs from the spelling provided in the article metadata: D. González-Cuadra, D. Bluvstein, M. Kalinowski, R. Kaubruegger, N. Maskara, P. Naldesi, T. V. Zache, A. M. Kaufman, M. D. Lukin, H. Pichler, B. Vermersch, Jun Ye, and P. Zoller. The spelling provided in the manuscript has been retained; please confirm. Although qubit-based quantum computers can potentially tackle this problem more efficiently than classical devices, encoding nonlocal fermionic statistics introduces an overhead in the required resources, limiting their applicability on near-term architectures. In this work, we present a fermionic quantum processor, where fermionic models are locally encoded in a fermionic register and simulated in a hardware-efficient manner using fermionic gates. We consider in particular fermionic atoms in programmable tweezer arrays and develop different protocols to implement nonlocal gates, guaranteeing Fermi statistics at the hardware level. We use this gate set, together with Rydberg-mediated interaction gates, to find efficient circuit decompositions for digital and variational quantum simulation algorithms, illustrated here for molecular energy estimation. Finally, we consider a combined fermion-qubit architecture, where both the motional and internal degrees of freedom of the atoms are harnessed to efficiently implement quantum phase estimation as well as to simulate lattice gauge theory dynamics.

## Significance

Neutral atoms trapped in tweezer arrays have recently emerged as powerful quantum simulation platforms, with recent experiments targeting quantum spin models. In this work, we envision the next generation of programmable atomic quantum simulators, where not only the atom’s internal but also motional degrees of freedom are controlled to process quantum information. In the case of fermionic atoms, this allows to encode and simulate fermionic models locally, where Fermi statistics are guaranteed at the hardware level. We develop a set of fermionic quantum gates acting on this fermionic register, including digital tunneling gates, and use it to construct fermionic circuits. This approach reduces circuit depths for quantum simulation significantly compared to qubit encodings, which always incur resource overheads.

The study of strongly correlated fermionic systems lies at the core of some of the most interesting problems in modern physics. These include profound questions regarding the inner workings of the universe, such as the physics of quark–gluon plasmas (1), as well as technologically pressing challenges in material science and quantum chemistry, from high-temperature superconductivity (2) to nitrogen fixation (3). The defining feature of fermionic many-body systems is the fundamental indistinguishability of their constituents, which dictates the antisymmetry of the wavefunction and the corresponding quantum statistics. Importantly, this indistinguishability gives rise to the so-called sign problem, which severely limits the applicability of many numerical approaches, such as Monte Carlo methods, highlighting the innate difficulty of solving fermionic many-body problems on classical computers (4).

One of the most promising alternatives to address these problems is provided by quantum computers (5). Traditionally, quantum computing involves distinguishable spin-1/2 particles, where quantum information is stored in superposition states of qubit registers and processed by the action of quantum gates. The quantum statistics of fermions needs to be encoded in such qubit-based devices on a software level, which incurs overhead in circuit depths (6–9) or qubit numbers (10–12). This presents a substantial challenge for current experiments where noise limits gate and readout fidelities. Although this approach has been applied to simple fermionic models from quantum chemistry (13–18), condensed-matter (19–28), and particle physics (29–31), using quantum processors based on superconducting circuits or trapped ions, experimental studies have so far been restricted to small system sizes.

Neutral atom systems provide a route to bypass this issue and construct quantum devices where fermionic statistics are built-in on a hardware level. The natural indistinguishability of atoms, which come as bosons or fermions, is for instance leveraged in celebrated analog quantum simulations of Hubbard models in optical lattices (32–34). Recently, optical tweezers have emerged as powerful tools to trap and manipulate neutral atoms with an unprecedented level of programmability and scalability (35–43). So far, these systems have, however, mainly been used to realize spin models with distinguishable constituents (44–51), where each atomic position is pinned to a specific tweezer, internal electronic or nuclear spin states are used to represent qubit states, and interactions between these qubits are implemented using highly excited Rydberg states.

In this work, we envision the next generation of such tweezer setups, where not only the internal but also the external degrees of freedom are coherently controlled and fully integrated in the quantum processing architecture. This is a crucial prerequisite for capitalizing on the indistinguishability of (fermionic) atoms, which requires the possibility for their center-of-mass wave functions to overlap, e.g., by coherently delocalizing atoms across tweezers. Remarkably, this motional control has already been demonstrated in pioneering proof-of-principle experiments both with tweezers and double-well optical potentials (52–59). Below, we describe a blueprint of the elements required for such a fermionic quantum processor (8), where both quantum hardware and software are codesigned to efficiently simulate fermionic models. More precisely, we describe protocols for the basic set of fermionic quantum gates, including, apart from Rydberg-mediated interacting gates, digital tunneling gates (or “fermionic beam splitters”) implemented through MERGE or SHUTTLE protocols. While the present work focuses on a setup with tweezer arrays, it is worth mentioning alternative setups that involve optical lattices, developed in the context of bosonic atoms (60–67). We exemplify our proposal for concrete atomic systems and discuss the requirements and experimental challenges for their physical implementation. Furthermore, we provide illustrative examples of application of such a fermionic quantum processor in the context of digital quantum simulation for quantum chemistry and for lattice gauge theories (LGT), where we use this fermionic gate set to find efficient circuit decompositions and demonstrate a significant reduction in circuit depth when compared to traditional qubit-based approaches.

## Hardwired Fermi Statistics

**Fig. 1.**
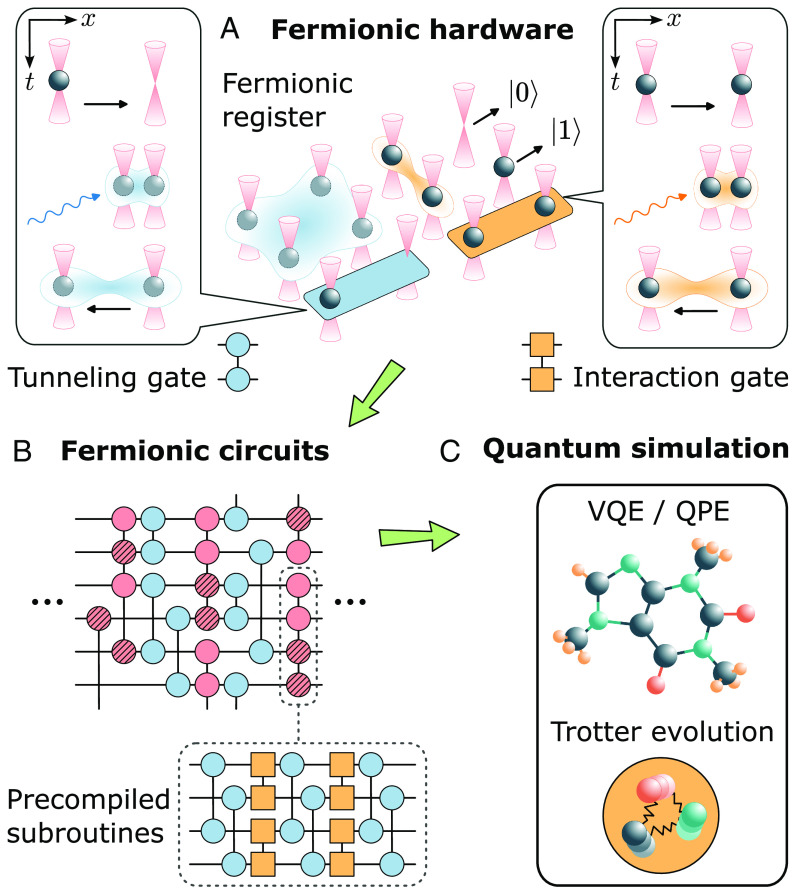
Fermionic quantum processor. (*A*) We consider a fermionic register based on fermionic atoms trapped in optical tweezers, where quantum information is encoded in the atomic occupation and processed using fermionic gates. The latter includes tunneling processes, delocalizing atoms between different tweezers (lighter spheres), as well as interaction gates, based on the Rydberg blockade mechanism. (*B*) We use these gates to construct fermionic quantum circuits, where certain subroutines are first precompiled to minimize circuit depths (as detailed below). (*C*) Fermionic circuits are particularly suited for quantum simulation of fermionic models, avoiding nonlocal overheads. Here, we consider the ground-state energy estimation of molecules using variational algorithms, as well as Trotter time evolution of LGTs.

We consider fermionic atoms in an array of microtraps that represent the fermionic quantum register (Fig. 1*A*). We write $c_{j,\sigma }$ ($c_{j,\sigma }^{†}$) for the annihilation (creation) operator of atoms on lattice site j, which we assume to be prepared in the trap ground state, and σ labeling internal atomic states [qudits (68)]. To be concrete, we will illustrate the quantum gate set, including in particular digital tunneling gates, for spinless fermions, i.e., dropping the index σ for the moment. In this case, the state of the quantum register comprising N atoms occupying L microtraps is given by a superposition of Fock states $|n_{1},\ldots ,n_{L}\rangle$, where $n_{j}=0,1$ is the atomic occupation, and $\sum_{j=1}^{L}n_{j}=N$.

In the context of quantum simulation (QS) of many-body systems (5), we are interested in particle-number conserving unitaries acting on this register (69), which can be constructed using the gate set

[1] $$\begin{array}{c} \mathrm{BK}=\left\{e^{i\pi /4n_{i}},\;e^{i\pi n_{i}n_{j}},\;e^{i\pi /4\left(c_{i}^{†}c_{j}+\;\text{H.c.}\right)}\right\}, \end{array}$$

as shown by Bravyi and Kitaev (8).

As we demonstrate in the next section, the circuit depth required to simulate fermionic models can be considerably shortened by considering instead the more general set $G=\left\{U_{i,j}^{\left(\mathrm{int}\right)}\left(\theta \right),\;U_{i,j}^{\left(t\right)}\left(\vec{\theta }\;\right)\right\}$, where

[2] $$\begin{array}{cc} U_{i,j}^{\left(int\right)}\left(\theta \right) & \equiv e^{-i\theta \;n_{i}n_{j}}\;, \end{array}$$

[3] $$\begin{array}{cc} U_{i,j}^{\left(t\right)}\left(\vec{\theta }\;\right) & \equiv e^{-i\left[\frac{\theta_{1}}{2}\left(e^{-i\theta_{2}}c_{i}^{†}c_{j}+\;\text{H.c.}\right)+\frac{\theta_{3}}{2}\left(n_{i}-n_{j}\right)\right]}, \end{array}$$

are generalized interaction (int) and tunneling (t) gates, respectively (Fig. 1*A*). The gates are parametrized in terms of tunable parameters θ and $\vec{\theta }=\left(\theta_{1},\theta_{2},\theta_{3}\right)$. In the context of qubit-based quantum computation, where single-qubit rotations together with one entangling interaction gate are sufficient to achieve universality (70), fermionic degrees of freedom first need to be encoded into qubits using, e.g., a Jordan–Wigner (JW) transformation (71). Tunneling gates, required to simulate many-body fermionic systems, can be implemented in the case of a JW encoding using O(L) entangling gates (71). Fermionic atoms trapped in the motional ground state of optical tweezers offer the unique possibility to avoid this overhead by implementing the gate set G directly. Specifically, the tunneling gate, $U_{i,j}^{\left(t\right)}\left(\vec{\theta }\;\right)$, can be realized using different approaches that exploit the capability of dynamically rearranging the tweezer positions, two of which we discuss now in more detail.

**Fig. 2.**
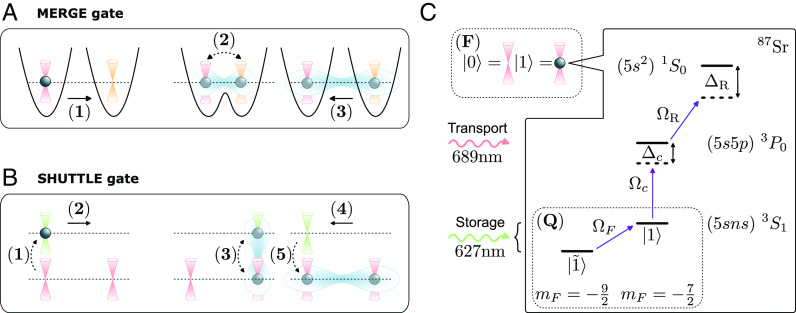
Fermion-qubit register and tunneling gates. (*A* and *B*) show the sequence of laser pulses and tweezer moves used to implement a MERGE and a SHUTTLE gate between a pair of sites (i,j), respectively, where the atomic superposition between two different tweezers is achieved either through direct tunneling (dashed line) or internal rotations. In the figure, tweezers of different colors correspond to different wavelengths. While the illustration shows the case of a single initially localized atom, we emphasize that the protocols also apply to situations where both tweezers contain contributions from many-body superpositions of several atoms delocalized over the whole system. (*C*) Level structure of ${}^{87}\mathrm{Sr}$. A fermionic register (**F**) is built by encoding quantum information into the presence/absence of an atom trapped by a given storage tweezer (red) in one of the hyperfine states of the ground-state manifold ${}^{1}S_{0}$. The latter is laser-coupled to the meta-stable excited state ${}^{3}P_{0}$, with Rabi frequency $\Omega_{c}$ and detuning $\Delta_{c}$, trapped by a second transport tweezer (green). Interactions between pairs of atoms are turned on by exciting the atom to the Rydberg state ${}^{3}S_{1}$, using a Rabi frequency $\Omega_{R}$, where $\Delta_{R}$ is the corresponding detuning. Other hyperfine levels, energy resolved using a magnetic field and coupled through a microwave frequency $\Omega_{F}$, serve as a qubit register (**Q**).

The MERGE approach realizes the tunneling gate by temporarily bringing the two tweezers i and j so close together that atoms can tunnel between the corresponding lowest vibrational states (Fig. 2*A*), as it has been demonstrated in recent experiments (52, 56–58, 72). Another approach involves fully merging (and later separating) the tweezer pair through custom-designed merging and splitting protocols. The gate parameters $\vec{\theta }$ can in either case be completely controlled by the tweezer parameters and the details of the merging protocol, such as the tweezer detuning and depths, the time-dependent distance of the tweezer minima, and the duration of this coupling process. In practice, these can be determined via optimal control techniques for given target tunnel parameters $\vec{\theta }$ and allow gate execution on timescales set by the inverse trapping frequency of the tweezers. This approach is therefore natural for light atoms, such as lithium, where relatively small trap depths are sufficient for large trap frequencies. We note that the implementation of the tunneling gate using the MERGE protocol requires the use of two different laser frequencies for the tweezer pair, to avoid heating processes arising from the interference across tweezers (42) (Fig. 2*A*).

The SHUTTLE approach offers an alternative way to realize the tunneling gate, which is based on the capability to realize state-dependent optical potentials (63, 73), and is thus naturally suited for alkaline-earth atoms. We therefore illustrate this idea for the specific example of ${}^{87}\mathrm{Sr}$, a fermionic isotope of strontium with nuclear spin I=9/2 below and in Fig. 2. The central idea is to use two sets of tweezers: a set of static storage traps, whose occupations define the fermionic register and a set of transport traps, which serve as a “shuttle” for atoms. Crucially, the wavelength of the storage and transport tweezers is chosen such that they trap two different internal states of an atom, respectively. For instance, for ${}^{87}\mathrm{Sr}$, one can trap the state ${}^{1}S_{0}$ (74) in the storage tweezers, and independently trap the clock state ${}^{3}P_{0}$ (63, 75) in transport tweezers, reminiscent of the collisional entangling quantum gate with spin-dependent lattices for bosonic atoms (60, 62, 67). Here, we extend these ideas and design a fermionic shuttle that implements the tunneling gate $U_{i,j}^{\left(t\right)}\left(\vec{\theta }\;\right)$. Importantly, when a storage and transport tweezer overlap and their potential shapes match, atoms can be coherently transferred or coherently split between the two tweezers simply by using laser pulses that implement internal rotations $R_{i}\left(\vec{\theta }\;\right)=e^{-i\left[\frac{\theta_{1}}{2}\left(\cos\;\theta_{2}X+\sin\;\theta_{2}Y\right)+\frac{\theta_{3}}{2}Z\right]}$, where X, Y, and Z are Pauli matrices acting on the atomic subspace spanned by ${}^{1}S_{0}$ and ${}^{3}P_{0}$. Using this mechanism, one can construct the full tunneling gate, $U_{i,j}^{\left(t\right)}\left(\vec{\theta }\;\right)$, as follows (see also Fig. 2*B*): 1) We first bring a transport tweezer to a storage site i and perform a π-pulse rotation, i.e., $R_{i}^{x}\left(\pi \right)\equiv R_{i}\left(\pi ,0,0\right)$, 2) after which we move the transport tweezer to site j. 3) We then perform a second pulse $R_{j}\left({\vec{\theta }}^{*}\right)$, with ${\vec{\theta }}^{*}=\left(\theta_{1},\theta_{2}+\frac{\pi }{2},\theta_{3}\right)$, 4) bring the transport tweezer back to site i, and 5) finally undo the initial π-pulse.

Contrary to the MERGE gate, there is no direct tunneling between tweezers involved in the SHUTTLE gate. Instead, the superposition between internal states created by the pulse at step (3) in the protocol translates to a superposition between different tweezers thanks to their state-dependent nature. The implementation time of this step depends thus on the Rabi frequency but is independent on the mass of the atom, allowing to implement the SHUTTLE gate on deep traps where unwanted motional excitations can be further suppressed. All timescales involved in this implementation are therefore small compared to moving times, such that the trap depth will set the clock speed of our fermionic quantum processor. Finally, we note that both the SHUTTLE and the MERGE approach can be fully parallelized. We comment on potential error sources for both approaches below.

In these setups, the interaction gate $U_{i,j}^{\left(\mathrm{int}\right)}\left(\theta \right)$ is essentially equivalent to a standard qubit entangling gate that has already been implemented for alkaline (35, 38, 39, 42) as well as alkaline-earth atoms (40, 76) using the Rydberg blockade mechanism. To realize it, we first rearrange the tweezers i and j and bring them to a distance that lies within the Rydberg blockade radius. We then drive the atoms with a laser that couples the atoms’ internal state to a Rydberg state. Owing to the Rydberg blockade mechanism, properly chosen laser pulses result in a unitary $e^{i\phi_{01}\left(n_{i}+n_{j}\right)+i\phi_{11}n_{i}n_{j}}$ (38). The phases $\phi_{01/11}$ depend on the shape of the Rydberg laser pulse (38) and choosing $\phi_{11}=2\phi_{01}-\theta$ provides the desired interaction gate up to the single-particle phase shift $\phi_{01}$. We note that resonantly coupling the atom to the untrapped Rydberg state could create excitations in the trap, which should be suppressed to preserve the atom’s indistinguishability. We note that these effects can be further reduced by trapping the atoms also in the Rydberg state (77). Alternatively, one can work in the dressed-Rydberg regime, where decay from the Rydberg state is suppressed and interactions become independent of distance, thus avoiding the repulsive forces between atoms. Below, we will discuss these together with other experimental challenges, including several strategies to mitigate the dominant error sources.

## Fermionic Quantum Circuits

We now employ the set of fermionic gates G to construct the quantum circuits required for QS of fermionic systems. Let us first focus on purely fermionic Hamiltonians, which we generalize below to fermion-boson models relevant to high-energy physics. To be explicit, we consider the particle-number-conserving fermionic Hamiltonian:

[4] $$\begin{array}{cc} H & =\sum_{i,j}h_{i,j}^{\left(1\right)}c_{i}^{†}c_{j}+\sum_{i,j,k,l}h_{i,j,k,l}^{\left(2\right)}\;c_{i}^{†}c_{j}^{†}c_{k}c_{l}\;, \end{array}$$

with complex parameters $h_{\mathrm{ij}}^{\left(1\right)}$ and $h_{\mathrm{ijkl}}^{\left(2\right)}$. This Hamiltonian is frequently used both in condensed matter (32) and quantum chemistry (71), where the indices can denote either the position of electrons in a solid-state crystal or the orbitals of a molecule, respectively. We note that spinfull fermionic models such as the Hubbard model can be recast into the form of Eq. **4** by mapping spin states to distinct spatial indices. These indices are encoded into separate tweezers within the fermionic register which are then encoded into different tweezers in the fermionic register. Specifically, we can represent the Hubbard interaction $n_{i,↑}n_{i,↓}$ as $n_{i_{1}}n_{i_{2}}$, where $i_{1}$ and $i_{2}$ correspond to two different tweezers, for direct implementation using the aforementioned interaction gate.

Many QS algorithms, e.g., the Variational Quantum Eigensolver (VQE) (13, 15) and the Quantum Approximate Optimization Algorithm (QAOA) (78), or Trotter time evolution (69), use as subroutines unitary operations obtained from exponentiating each local term in the Hamiltonian. Apart from the tunneling and interaction gates introduced above, these include density-dependent tunneling (dt) as well as pair-tunneling (pt) processes,

[5] $$\begin{array}{cc} U_{i,j,k}^{\left(\mathrm{dt}\right)}\left(\theta_{1},\theta_{2}\right) & \equiv e^{-i\;\theta_{1}\left(e^{-i\theta_{2}}c_{i}^{†}n_{j}c_{k}+\;\text{H.c.}\right)}, \end{array}$$

[6] $$\begin{array}{cc} U_{i,j,k,l}^{\left(\mathrm{pt}\right)}\left(\theta_{1},\theta_{2}\right) & \equiv \;e^{-i\;\theta_{1}\left(e^{-i\theta_{2}}c_{i}^{†}c_{j}^{†}c_{k}c_{l}+\;\text{H.c.}\right)}, \end{array}$$

where the indices in the equations above are different.

**Fig. 3.**
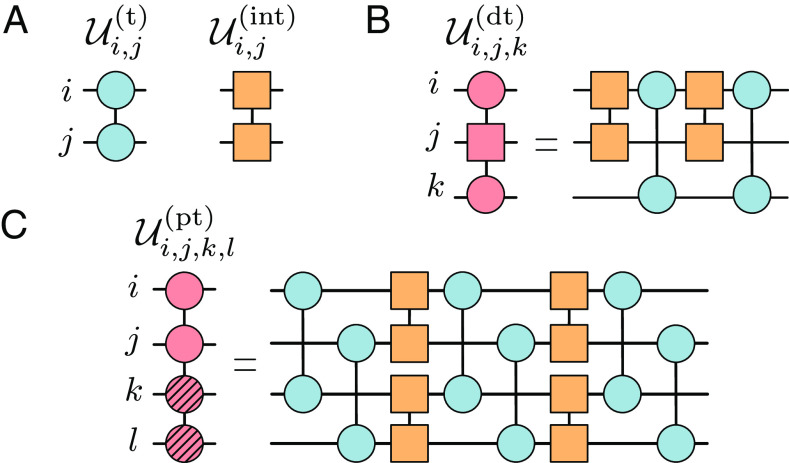
Fermionic subroutines. In (*A*), we indicate the elementary fermionic gates, namely the tunneling and interaction gate. Using these elementary gates, we can construct more complex gates like a density-dependent tunneling gate (*B*) and a pair-tunneling gate (*C*) where a pair of fermions can tunnel together from the hatched to the unhatched sites and vice versa. The specific angles required for an exact circuit decomposition of these compiled gates are provided in Eqs. **7** and **9**.

For both Eqs. **5** and **6**, we derived a specific decomposition in terms of the gate set G, which are shown in Fig. 3 *B* and *C*, respectively. Both decompositions are exact and have a constant circuit depth that does not depend on the system size L. More precisely, for Eq. **5**, we obtain

[7] $$\begin{array}{c} \begin{array}{cc} U_{i,j,k}^{\left(\mathrm{dt}\right)}\left(\theta_{1},\theta_{2}\right) & =U_{i,k}^{\left(t\right)}\left(\theta_{1},\theta_{2},0\right)\;U_{i,j}^{\left(\mathrm{int}\right)}\left(\pi \right)\\ & \;\cdot U_{i,k}^{\left(t\right)}\left(-\theta_{1},\theta_{2},0\right)\;U_{i,j}^{\left(\mathrm{int}\right)}\left(\pi \right), \end{array} \end{array}$$

which can derived analytically by first expanding $U_{i,j,k}^{\left(\mathrm{dt}\right)}\left(\theta_{1},\theta_{2}\right)$ in its Taylor series and then using the relation

[8] $$\begin{array}{c} e^{-i\pi n_{l}n_{j}}\left(c_{l}^{†}c_{k}+\;\text{H.c.}\right)e^{i\pi n_{l}n_{j}}=c_{l}^{†}\left(1-2n_{j}\right)c_{k}+\;\text{H.c.}, \end{array}$$

Although a similar analytical decomposition can be obtained for Eq. **6**, here, we report on a more compact and still exact circuit decomposition, found via variational optimization. The latter, displayed in Fig. 3*C*, minimizes the total circuit depth and takes the following form:

[9] $$\begin{array}{c} \begin{array}{cc} U_{i,j,k,l}^{\left(pt\right)}\left(\theta \right) & =U_{\overset{i,k}{j,l}}^{\left(t\right)}\left(\frac{\pi }{2},\frac{\theta_{2}+2\pi }{2},0\right)\;U_{\overset{i,j}{k,l}}^{\left(int\right)}\left(\theta_{1}\right)\\ & \;\cdot U_{\overset{i,k}{j,l}}^{\left(t\right)}\left(\frac{\pi }{2},\frac{\theta_{2}+\pi }{2},0\right)\;U_{\overset{i,j}{k,l}}^{\left(int\right)}\left(-\theta_{1}\right)\;\\ & \;\cdot U_{\overset{i,k}{j,l}}^{\left(t\right)}\left(\sqrt{2}\alpha ,\frac{2\theta_{2}-\pi }{4},\alpha \right), \end{array} \end{array}$$

where we denote $U_{\overset{i,k}{j,l}}^{\left(t\right)}\left(\vec{\theta }\;\right)\equiv U_{i,k}^{\left(t\right)}\left(\vec{\theta }\;\right)\;U_{j,l}^{\left(t\right)}\left(\vec{\theta }\;\right)$ and $U_{\overset{i,j}{k,l}}^{\left(\mathrm{int}\right)}\left(\theta \right)\equiv U_{i,j}^{\left(\mathrm{int}\right)}\left(\theta \right)\;U_{k,l}^{\left(\mathrm{int}\right)}\left(\theta \right)$, with the two gates applied in parallel, and $\alpha =2\pi /\sqrt{27}$.

These fermionic circuits in terms of the gate set G provide a clear advantage in terms of circuit depths with respect to qubit encodings, which necessarily suffer from overheads associated with implementing nonlocal fermionic statistics. Such encodings often introduce space overhead with ancillary degrees of freedom (10–12), or depth overhead caused by the decomposition of significantly higher-weight qubit operators (6–9, 79), resulting from mapped fermionic operators; for the case of Jordan–Wigner transformation, the size of such qubit terms grows with the system size L (71). Moreover, the ability to implement tunneling and interaction gates at arbitrary angles enables us to find exact decompositions, resulting in shorter quantum circuits compared to approximate decompositions based on the universal BK set [**1**]. Finally, note that the all-to-all connectivity of atom arrays plays a crucial point in the case of quantum chemistry, where the presence of nonlocal terms in Eq. **4** introduces further overhead for architectures with only nearest-neighbor gates (80).

The fermionic quantum circuits can subsequently serve as precompiled subroutines, thereby extending the polynomial savings in circuit depth to the entire QS algorithm. For instance, the real-time evolution under Hamiltonian Eq. **4** can be simulated by a first-order Trotter expansion (81),

[10] $$\begin{array}{c} e^{-iHt}\approx {\left[\prod_{i,j}U_{i,j}^{\left(t\right)}\left(h_{i,j}^{\left(1\right)}\delta t,0,0\right)\prod_{i,j,k,l}U_{i,j,k,l}^{\left(\mathrm{pt}\right)}\left(h_{i,j,k,l}^{\left(2\right)}\delta t,0\right)\right]}^{t/\delta t}, \end{array}$$

where δt is the Trotter time and the local gates can be applied in parallel across the system.

Another application is hybrid quantum-classical algorithms, such as VQE, which are specifically designed for determining the ground-state energy of molecules using near-term quantum devices. In this context, utilizing $U_{i,j,k,l}^{\left(\mathrm{pt}\right)}$ enables us to construct variational states that exhibit two important characteristics. First, they are hardware efficient (80) by directly utilizing the gates naturally available in the fermionic quantum processor. Second, these states possess what is known as “chemically inspired” properties (71), meaning they have sufficient expressiveness to efficiently approximate the relevant physical states within the entire Hilbert space.

One example is the disentangled unitary coupled cluster (dUCC) ansatz (82), $|\psi (\vec{\theta })\rangle =U(\vec{\theta })|\psi_{\text{HF}}\rangle$, constructed using the variational circuit

[11] $$\begin{array}{c} U\left(\vec{\theta }\right)=\prod_{i,\alpha }U_{i,\alpha }^{\left(t\right)}\left(\theta_{i,\alpha }^{\left(1\right)},\frac{\pi }{2},0\right)\prod_{i,j,\alpha ,\beta }U_{i,j,\alpha ,\beta }^{\left(\mathrm{pt}\right)}\left(\theta_{i,j,\alpha ,\beta }^{\left(2\right)},\frac{\pi }{2}\right), \end{array}$$

where the products run over occupied (i,j) and virtual (α,β) modes with respect to an initial Hartree–Fock product state $|\psi_{\text{HF}}\rangle$. The energy functional $E\left(\vec{\theta }\right)=\langle \psi \left(\vec{\theta }\right)|H|\vec{\psi }\left(\vec{\theta }\right)\rangle$, which is then classically minimized, can be also efficiently constructed in our fermionic processor, by first applying randomized tunneling gates followed by measurements in the occupation basis (83, 84).

**Fig. 4.**
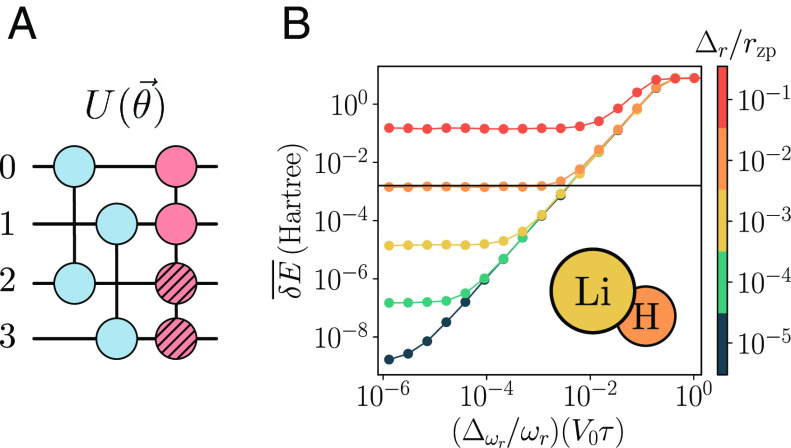
Variational circuit for VQE. (*A*) A variational circuit used to prepare the ground-state of the LiH molecule. (*B*) Average energy difference $\bar{\delta E}$ in the presence of fluctuations in the trapping frequency and tweezer positions, characterized by standard deviations $\Delta_{\omega_{r}}$ and $\Delta_{r}$, respectively. $V_{0}$, $\omega_{r}$, and $r_{\mathrm{zp}}$ denote the depth, radial frequency, and zero-point fluctuations of the harmonic trap, and τ is the transfer pulse time (Methods). The horizontal line signals chemical accuracy, $\delta E^{*}\approx 1.59$ mHa.

We illustrate our approach for the LiH molecule. We consider in particular the molecule at a fixed interatomic distance of 1.45 Å in the Born–Oppenheimer approximation, with two electrons and four active orbitals. The Hamiltonian parameters $h_{i,j}^{\left(1\right)}$ and $h_{i,j,k,l}^{\left(2\right)}$ for this configuration are obtained using OpenFermion (85). The circuit that prepares, in this case, the dUCC ansatz is depicted in Fig. 4*A*, where the gates $U_{i,\alpha }^{\left(t\right)}\left(\theta_{i,\alpha }^{\left(1\right)},\frac{\pi }{2},0\right)$ and subroutines $U_{i,j,\alpha ,\beta }^{\left(\mathrm{pt}\right)}\left(\theta_{i,j,\alpha ,\beta }^{\left(2\right)},\frac{\pi }{2}\right)$ that appear in the circuit correspond to the nonzero terms in the Hamiltonian.

We calculate the energy difference $\delta E\equiv E_{\exp}\left({\vec{\theta }}^{*}\right)-E_{0}$, where ${\vec{\theta }}^{*}$ are the optimal variational parameters that minimize $E\left(\vec{\theta }\;\right)$, $E_{\exp}$ is obtained by evaluating the latter in the presence of experimental errors, and $E_{0}$ is the exact ground-state energy. Fig. 4*B* shows the average $\bar{\delta E}$ for random fluctuations in the trapping frequency and tweezer positions (*Materials and Methods*), providing an estimate of the required precision to reach chemical accuracy. Note that $\bar{\delta E}$ can be reduced by lowering the tweezer depth as well as by increasing the pulse intensity to reduce the implementation time, and the full protocol including tweezer transport can be further improved using optimal control (86, 87).

In the last years, more advanced versions of VQE have been developed to address larger molecules using fewer resources. One example is adapt-VQE (88–90), where the variational circuits are iteratively constructed by progressively adding new layers built from an operator pool. The latter are chosen to maximize the reduction in energy at every iteration, and the procedure stops once a certain precision is achieved. One can again choose a chemically inspired operator pool, composed of the subroutines Eqs. **5** and **6**, where each of them requires again additional overhead in a qubit-based quantum computer (88). Alternatively, the number of entangling gates can be reduced by considering instead a hardware-efficient operator pool, e.g., by dropping the Pauli strings in the JW transformation as in qubit-adapt-VQE (89), which however further complicates the classical optimization part of the algorithm. Again, our fermionic quantum processor combines the best of these two approaches, as it allows us to construct each element in the operator pool with a constant-depth circuit while minimizing the complexity of the classical optimization.

## Fermion-Qubit Architecture

We now consider spinfull fermionic atoms, combining the fermionic register and fermion gates introduced above with a more standard qubit-based architecture. This allows us to encode both qubit ancillas and fermionic modes locally, leading to efficient implementations of more advanced QS algorithms such as quantum phase estimation (QPE) (91), as well as to simulate boson-fermion models such as LGTs in a hardware-efficient manner.

**Fig. 5.**
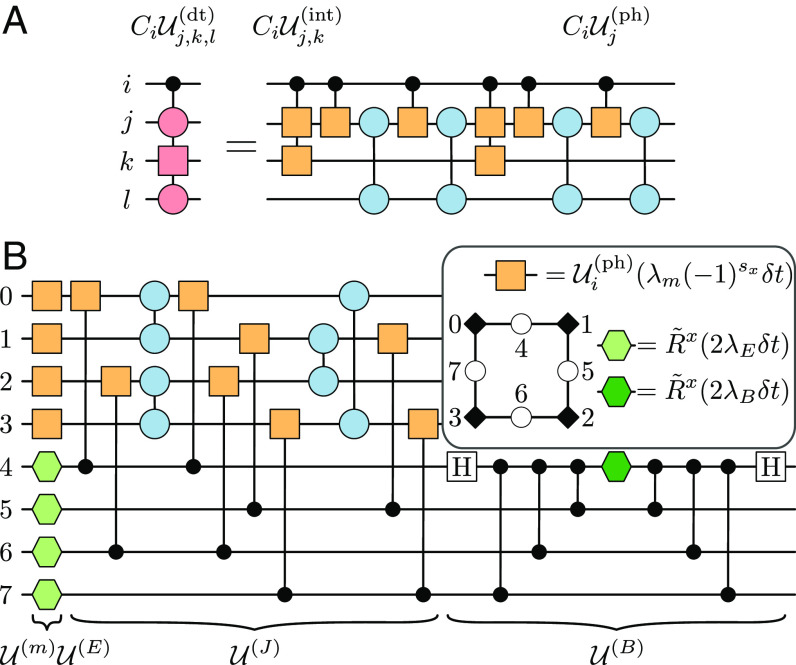
Fermion-qubit quantum circuits. (*A*) Decomposition of $C_{i}U_{j,k,l}^{\left(\mathrm{dt}\right)}$$\left(\theta_{1},\theta_{2}\right)$, a density-dependent fermionic hopping controlled by ancillary qubit (i) into fermionic gates and the three-body fermion-qubit gate $C_{i}U_{j,k}^{\left(\mathrm{int}\right)}$. The angles of the interaction and the controlled interaction gate are π. The angles of the first and third tunneling gates are $\left(\theta_{1},\theta_{2},0\right)$, whole for the second and fourth are $\left(\theta_{1},-\theta_{2},0\right)$. (*B*) Trotter step required to time-evolve one plaquette under the $Z_{2}$ LGT Hamiltonian Eq. **12**), where atoms 0 to 4 and 5 to 7 encode local matter and gauge fields, respectively. The unitary circuits $U^{\left(p\right)}$, with p∈{m,E,J,B}, implement the exponential of each term in the Hamiltonian, and the single-qubit rotations are given by ${\tilde{R}}^{x}\left(\theta \right)=e^{-i\left(\theta /2\right)\sigma^{x}}$ (green hexagons) acting on the $\left\{|\tilde{1}\rangle ,|1\rangle \right\}$ subspace, while $U_{i}^{\left(\mathrm{ph}\right)}\left(\theta \right)=e^{-i\theta n_{i}}$ (orange box) is a local phase shift if a particle is present in tweezer i. We further use standard notations for qubit operations, namely, H indicates a Hadamard gate $H=(-|1\rangle +|\tilde{1}\rangle )\langle 1|/\sqrt{2}+(|1\rangle +|\tilde{1}\rangle )\langle \tilde{1}|/\sqrt{2}$, a dot denotes a control on the state $|1\rangle$, and two connected dots correspond to a CZ gate. The angles of the interaction gates are π, whereas the angles of the tunneling gates are $\left(\lambda_{J}\delta t,0,0\right)$.

To be specific, we illustrate the architecture using again Sr as an example. Qubit ancillas can be readily included by considering two hyperfine levels of ${}^{1}S_{0}$ (Fig. 2*C*), where one level, denoted $|\tilde{1}\rangle$, is decoupled from the ${}^{3}P_{0}$ clock manifold by a magnetic field. Gates in G applied to an atom in the internal state $|\tilde{1}\rangle$ act therefore as the identity, similarly to the empty state $|0\rangle$. Defining $\sigma^{z}=|\tilde{1}\rangle \langle \tilde{1}\left|-|1\rangle \langle 1\right|$ and $\sigma^{x}=|1\rangle \langle \tilde{1}\left|+\right|\tilde{1}\rangle \langle 1|$, we can implement corresponding rotations $\tilde{R}$ between the states $|\tilde{1}\rangle$ and $|\tilde{1}\rangle$ through microwave frequencies (Fig. 2*C*). This enlarges the gate set to $\tilde{G}$, including both G and $\tilde{R}$. In the fermion-qubit register, the same type of atoms can either encode a qubit ancilla or a fermionic mode. In the case of QPE, the energy of H can be estimated by applying a Trotter time evolution under the terms in H controlled by an ancilla, with a precision that grows with the number of ancillas (91). As an example, we show in Fig. 5*A* a fermion-qubit circuit associated to a controlled density-dependent hopping process $C_{i}U_{j,k,l}^{\left(\text{dt}\right)}(\theta_{1},\theta_{2})=|\tilde{1}\rangle {\langle \tilde{1}|}_{i}⊗I_{j,k,l}+|1\rangle {\langle 1|}_{i}⊗U_{j,k,l}^{\left(\text{dt}\right)}(\theta_{1},\theta_{2})$, and other controlled operations can be decomposed similarly in terms of our fermionic gate set. We note that this decomposition requires additional controlled phase gates $C_{i}U_{j,k}^{\left(\mathrm{int}\right)}\left(\theta \right)$ and $C_{i}U_{j}^{\left(\text{ph}\right)}(\theta )=|\tilde{1}\rangle {\langle \tilde{1}|}_{i}⊗I_{j}+|1\rangle {\langle 1|}_{i}⊗e^{-i\theta n_{j}}$, which can be directly implemented using the Rydberg blockade mechanism (38).

Finally, the fermion-qubit architecture allows us to go beyond purely fermionic models, and consider for instance LGTs, where fermions are coupled to dynamical (bosonic) gauge fields. Consider for simplicity a $Z_{2}$ LGT described by the Hamiltonian

[12] $$\begin{array}{c} \begin{array}{cc} H & =\lambda_{E}\sum_{\left\langle x,y\right\rangle }\sigma_{\left\langle x,y\right\rangle }^{x}+\lambda_{B}\sum_{□}\sigma_{□}^{z}\\ & \;+\lambda_{J}\sum_{\left\langle x,y\right\rangle }\left(c_{x}^{†}\sigma_{\left\langle x,y\right\rangle }^{z}c_{y}+\;\text{H.c.}\right)+\lambda_{m}\sum_{x}{\left(-1\right)}^{s_{x}}n_{x}, \end{array} \end{array}$$

where fermion (spin) operators, representing matter (gauge) degrees of freedom, act on the sites x (links ⟨x,y⟩) of a D-dimensional lattice. The first row in Eq. **12** contains the pure-gauge dynamics, including four-body plaquette operators $\sigma_{□}^{z}$ acting with $\sigma_{\left\langle x,y\right\rangle }^{z}$ on each link around a plaquette □ (Fig. 5*B*, while the second row includes gauge–matter interactions. The Hamiltonian is invariant under local $Z_{2}$ transformation, i.e., $H=V_{x}^{†}HV_{x}$∀x, with $V_{x}={\left(-1\right)}^{n_{x}}\prod_{y}\sigma_{\left\langle x,y\right\rangle }^{x}$.

Apart from serving as simplified models to study fermionic confinement (92, 93), $Z_{2}$ LGTs emerge in condensed-matter systems (94), displaying strongly correlated phenomena such as high-Tc superconductivity (95), topological order (93, 96–98), and unconventional dynamics (99–101). For D>1, the model presents a sign problem away from half-filling and can not be solved efficiently using classical methods. The corresponding real-time dynamics can be efficiently simulated using quantum devices (102–107) through, e.g., a first-order Trotter expansion. In our fermion-qubit architecture, the gate set $\tilde{G}$ allows us to construct Trotter steps with a constant circuit depth. This can be seen by first decomposing the exponential of each term in the Hamiltonian Eq. **12** following refs. 108–110, where the corresponding circuit in terms of the fermionic and qubit gates in $\tilde{G}$ is shown in Fig. 5*B*. Thanks to the local structure of Eq. **12**, the possibility to parallelize fermionic gates and in particular the possibility to directly implement fermionic tunneling gates, such decomposition leads to a circuit depth that is independent of the system size, contrary to more standard qubit-based approaches. For the latter, any JW encoding will convert an extensive number of local fermionic terms to multiqubit operators acting each of them on an extensive number of qubits. Implementing the exponential of each of them would require therefore a number of two-qubit gates that also grows linearly with the system size. The total circuit depth for a single Trotter step will be thus polynomial in L, where the advantage of using instead fermionic gates becomes clear.

Finally, we note that the above protocol can be generalized to nonabelian gauge fields, required to address the full Standard Model of particle physics, by further extending it to a fermion-qudit architecture (110, 111).

## Experimental Challenges and Considerations

We finish by discussing in more detail some of the experimental challenges that should be overcome to build a fermionic quantum processor, including the main error sources for the gates discussed above, as well as different strategies to minimize them. The experimental setup for our proposed fermionic quantum processor is similar to existing reconfigurable tweezer platforms with high-fidelity Rydberg gates (42, 76); however, the main new challenge will be coherently controlling the motional degrees of freedom. This introduces new pathways for decoherence, primarily coming from leakage out of the fermionic register (i.e., heating to motional excited states), and dephasing from inhomogeneity between different tweezer sites.

Leakage, or heating, out of the motional ground state can arise from state preparation errors, scattering by the tweezer, pulsing the traps off for the Rydberg gate, and from moving the atoms; we argue here that all of these effects can be greatly suppressed. First, heating from tweezer scattering is negligible for sufficiently large detunings. By utilizing tight radial and axial confinement, 3D motional ground state preparation has been realized at levels of ≈95% (52, 57). However, by “spilling out” all motional excited states, we can convert the motional ground state occupancy to nearly 100% provided we can nondestructively check for atom presence, which can be done directly using an ancilla atom and a high-fidelity Rydberg gate to check for atom presence (112, 113).

Consider for example the 50-kHz trap depth for the lithium tweezers, relevant for the MERGE gate, used in refs. 56 and 58, which would give a 0.03-Hz scattering rate and an even smaller heating rate due to the Lamb-Dicke parameter. Pulsing the trap off during the Rydberg gate could cause heating, but the probability of transitioning to higher motional states will be roughly ${\left(\omega t\right)}^{2}/4$, where ω=2π×15 kHz is the trap frequency (56, 58) and t≈100 ns is the implementation time required for 99.9% fidelity gates. This probability then evaluates to <10${}^{-4}$ per gate and so does not contribute significantly compared to a 99.9% gate fidelity. Finally, moving the atoms can cause heating and, as a conservative estimate, we utilize the heating rates calculated in ref. 42. If the atoms are placed relatively close together, at a distance of several micrometers, then for the move to be 99.9 to 99.99% fidelity, each move has to be ≈500 μs. This can be significantly speed-up using optimal control (114).

In general, we expect that trap inhomogeneities will be the dominant source of dephasing for degrees of freedom coherently encoded in motional states. With a Rydberg gate fidelity of ∼99.9%, we would like to perform ∼1,000 operations, where in general, the tweezer geometry is reconfigured between each round. The latter would allow us to implement, e.g., a few Trotter steps required to simulate the real-time dynamics of local Hamiltonians, such as the LGT described above, for 2D systems with ∼100 atoms, an extremely challenging problem to tackle with a classical computer. If it takes ∼500 μs to move atoms between gates, that sets the total operation time of ∼500 ms. For the example of lithium-6, a trap depth of 50 kHz and a reasonable SD between tweezers of 0.2% leads to a coherence time of $T_{2}^{*}\approx 2$ ms, which is only enough time for ≈4 moves. Note that this is for shallow trap depths of lithium-6, a light atom and that this effect will be even more exacerbated for heavier atoms like strontium, where larger trap depths (laser intensities) are required for the same trap frequencies.

**Fig. 6.**
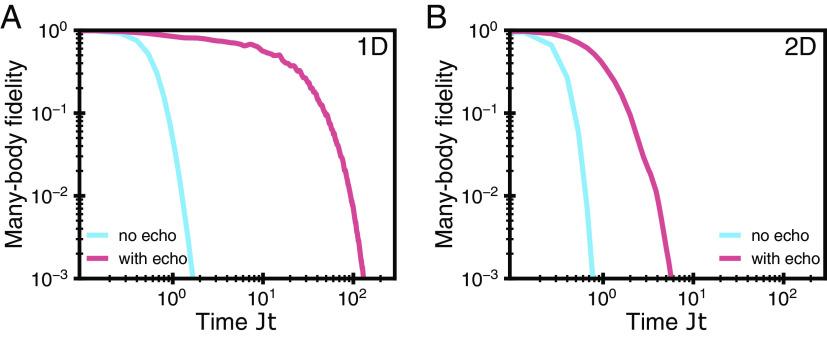
Robustness to trap inhomogeneity. (*A*) The achievable physical time in digital simulation is extended by two orders of magnitude when utilizing the positional echo procedure in one dimension. There, it is especially effective as the relative phase noise becomes bounded (*Materials and Methods*). The simulation was performed for a simple hopping Hamiltonian with periodic boundary conditions, a hundred sites, and a Gaussian phase disorder with $\sigma_{\theta }$=0.035 applied between tunneling events (see main text and *Materials and Methods*). (*B*) For a generic many-body system, the phase noise is no longer constant, but the echo procedure still improves coherence of the system. For a 10 × 10 square lattice, under the same conditions as the one-dimensional case, the available simulation time is extended by an order of magnitude.

Whenever there is static inhomogeneity in a system, such as positional trap-depth dependence, the natural approach is to perform a motional echo procedure (115). We can permute around the various tweezer positions so that all atoms acquire the same average phase due to the positional trap depth dependence. First, consider two tweezers with unequal trap intensities and thus unequal energies at the bottom of the trap. Due to the different energies, the atoms at these sites experience dephasing. However, this effect can be canceled out by repeatedly swapping the tweezer positions along with each application of the tunneling gate (see *Materials and Methods* for details). In Fig. 6, we illustrate this idea by simulating the Floquet evolution of two example Hamiltonians with nearest-neighbor hopping (*Materials and Methods*). The results show that performing the echo procedure allows us to extend the useful simulation time by two orders of magnitude in one dimension and by one order of magnitude in two dimensions. In practice, the inhomogeneities might not be exactly static, in which case the echo needs to be applied at a rate faster than that timescale. We also note that there could potentially be other methods to design robust sequences of tunneling gates. For example, circuits can be precompiled in order to minimize the number of necessary swap operations while taking into account the specific distribution of spatial coherence.

In addition, trap inhomogeneities can be further reduced by storing the atoms in state-dependent optical lattices in combination with tweezers, which are used to move the atoms to the desired position in order to apply the corresponding gates (60–67). In this architecture, the MERGE gate can be implemented using superlattices, by first placing the atoms in double wells (116), while the SHUTTLE gate can be implemented by using the lattice and tweezers as storage and transport potentials, respectively.

Finally, for the SHUTTLE gate based on alkaline-earth atoms like strontium as discussed above, additional error sources include phase shifts and losses during step (3) of the protocol due to elastic and inelastic collisions, respectively, between atoms in the ${}^{3}P_{0}$ and ${}^{1}S_{0}$ states. The formers are of the order of a few kHz for ${}^{87}$Sr (117, 118) and should be removed by an appropriate calibration or measured and compensated by additional phase shift gates. Inelastic collisions are even smaller (119) than typical Rabi frequencies and can therefore be safely neglected. Finally, we note that the optical potentials for the storage and transport tweezers need to match to properly transfer atoms between them, and fluctuations in the tweezer location and laser intensity will lead to imperfect transfer processes (*Materials and Methods*). The effect of such fluctuations is taken into account in the variational preparation of a simple molecule, illustrated in Fig. 3.

## Conclusions and Outlook

We presented a fermionic quantum processor based on fermionic atoms trapped in tweezer arrays and showed how to locally encode and quantum simulates fermionic models in a hardware-efficient manner. We illustrated the advantages of our approach with respect to qubit-based devices for VQE and QPE in quantum chemistry, as well as for the Trotter time evolution of gauge theories. We note that the proposed hardware can be used to run more advanced fermionic quantum algorithms, further optimizing the required resources, with the goal of reaching a practical quantum advantage in the near term. The fermionic gate set can also be extended to include particle nonconserving processes, using, e.g., atom reservoirs, allowing for the implementation of error correction protocols.

## Materials and Methods

### Error Model for VQE.

We exemplify the fermionic quantum simulation of the LiH molecule using VQE, where we consider in particular the tunneling gate implemented using the SHUTTLE protocol in strontium. We consider optical tweezers generated by laser fields with the following intensity profile in the radial r and longitudinal z directions,

[13] $$\begin{array}{c} I\left(r,z\right)=I_{0}\left(\frac{w_{0}}{w_{z}}\right)e^{-2{\left(r/w_{z}\right)}^{2}}, \end{array}$$

with $w_{z}=w_{0}\sqrt{1+{\left(z/z_{R}\right)}^{2}}$, where $w_{0}$ and $z_{R}=\pi w_{0}^{2}/\lambda$ are the waist and Rayleigh length of the tweezer, respectively, and λ is the laser (trapping) wavelength. For a properly chosen wavelength, the latter gives rise to an AC Stark shift on a neutral atom, leading to an optical potential V(r,z)=−Re(α)/(2ϵ0c)I(r,z), where α is the atom polarizability for the corresponding energy level, $\epsilon_{0}$ is the permittivity of free space, and c is the speed of light. The trapping potential V(r,z) can be approximated around its minimum using a second-order Taylor expansion, leading to the following harmonic potential,

[14] $$\begin{array}{c} V\left(r,z\right)=V_{0}+\frac{1}{2}m\omega_{r}^{2}r^{2}+\frac{1}{2}m\omega_{z}^{2}z^{2}, \end{array}$$

where $V_{0}\equiv V\left(0,0\right)$, m is the mass of the atom, and the frequencies are given by $\omega_{r}=\sqrt{4V_{0}/\left(mw_{0}^{2}\right)}$ and $\omega_{z}=\sqrt{2V_{0}/\left(mz_{R}^{2}\right)}$. For typical experimental parameters, these frequencies are related by $\omega_{r}/\omega_{z}\approx 10$. Here, we consider random fluctuations both in the frequencies $\omega_{r}$ and $\omega_{z}$ and in the relative position between tweezers, given by δr and δz, following all these parameters independent Gaussian distributions.

These experimental fluctuations introduce errors in the fermionic quantum gates since they lead to imperfect rotations between the storage (S) and transport (P) tweezers. In particular, both the Rabi coupling and the detuning of a given pulse are modified due to the imperfect overlap between the wavefunctions, $\Omega \left(\delta r,\delta z\right)=\Omega_{0}f\left(\delta r,\delta z\right)$ and $\Delta \left(\delta r,\delta z\right)=\Delta_{0}f\left(\delta r,\delta z\right)$, with

[15] $$\begin{array}{c} f\left(\delta r,\delta z\right)=\int \;drdz\;2\pi r\;\psi_{S}^{*}\left(r,z\right)\psi_{P}\left(r+\delta r,z+\delta z\right), \end{array}$$

where we consider Gaussian wavefunctions for the ground state of the harmonic potential Eq. **14**,

[16] $$\begin{array}{c} \psi_{S/P}\left(r,z\right)=\frac{e^{-{\left(r/2r_{\mathrm{zp}}\right)}^{2}}}{{\left(2\pi r_{\mathrm{zp}}\right)}^{1/4}}\frac{e^{-{\left(z/2z_{\mathrm{zp}}\right)}^{2}}}{{\left(2\pi z_{\mathrm{zp}}\right)}^{1/4}}, \end{array}$$

with zero-point fluctuations given by $r_{\mathrm{zp}}=1/\sqrt{2m\omega_{r}}$ and $z_{\mathrm{zp}}=1/\sqrt{2m\omega_{z}}$. Moreover, fluctuations in the trap frequency lead to a nonzero difference between the trap depths, $\delta V_{0}=V_{0}^{S}-V_{0}^{P}$, introducing an extra unwanted detuning, $\tilde{\Delta }=\Delta +\delta V_{0}$, giving rise to an extra angle $\delta V_{0}\tau$ for Z rotations, where τ is the pulse time.

### Motional Echo Scheme.

The positional trap-depth dependence leading to a static dephasing can be mitigated by an appropriate echo procedure. Due to the positional nature of this noise, the natural approach is to ensure that each fermionic register spends equal time at each tweezer site and, on average, experiences the same disorder pattern. The key insight is that, once the evolution is digital, the atom i does not need to always reside in the tweezer site i. That is, we can permute the atoms around the various tweezer positions either by moving them around or introducing swap operations, which effectively change which atom resides in which tweezer; since this process is deterministic, it is easy to keep track of the atom labels in the classical experimental software. Ideally, the atoms need to be shuffled in an ergodic fashion, such that they spend equal time at every tweezer site, ensuring the dephasing between two tweezers is reduced on average.

More concretely, during the t-th time step, the i-th atom resides in the $\sigma_{t}\left(i\right)$-th tweezer. Since the inhomogeneity is (to first-order) static, but varies from tweezer to tweezer, this can be modeled via a time-dependent disorder Hamiltonian $D_{t}=\sum_{x}h_{\sigma_{t}\left(x\right)}{\hat{n}}_{x}$.

The effective dynamics is captured by alternating disorder evolution and target evolution. Moving into an interaction picture with respect to the disorder, and assuming each moving step takes time τ, the hopping terms $c_{i}^{†}c_{j}$ become dressed as

[17] $$\begin{array}{cc} \left(c_{i}^{†}c_{j}\right)\left(t\right) & =e^{-i\tau \sum_{t^{\prime }\leq t}\left(h_{\sigma_{t^{\prime }}\left(j\right)}-h_{\sigma_{t^{\prime }}\left(i\right)}\right)}c_{i}^{†}c_{j}, \end{array}$$

where the term in the exponent leads to dephasing of the simulation; thus, if the atoms are not permuted between sites ($\sigma_{t}\left(j\right)=j$), the phase noise grows linearly in time. The echo procedure now consists of introducing a permutation scheme σ such that the error accumulation is reduced. We note the special nature of this echo procedure, which is intrinsically linked to spatial positions of the traps and cannot be performed by applying global operations, in contrast to the typical echo sequences present in spin systems.

There is a particularly simple and effective echo procedure for a 1D chain with periodic boundary conditions (j = i + 1). Here, the two sublattices of tweezers, labeled with even and odd numbers, will sequentially translate by one site in space after every tunneling gate resulting in $\sigma_{t}\left(i\right)=\left(i-t\right)\;\mathrm{mod}\;L$, where L is the length of the chain. This results in each atom spending the same amount of time at each position and, moreover, experiencing the same history of trap depths as the neighboring site, up to boundary conditions, which further improves the echo performance as the noise between the two sites becomes time-correlated (between Floquet rounds), $\sigma_{t+1}\left(i+1\right)=\sigma_{t}\left(i\right)$ and the relative accumulated disorder is

[18] $$\begin{array}{c} \sum_{t^{\prime }\leq t}\left(h_{\sigma_{t^{\prime }}\left(i+1\right)}-h_{\sigma_{t^{\prime }}\left(i\right)}\right)=h_{\sigma_{0}\left(i+1\right)}-h_{\sigma_{t}\left(i\right)}, \end{array}$$

which implies that the phase noise on the hopping term $t_{\mathrm{ij}}$ remains bounded for all time.

A more general disorder cancelation strategy is to simultaneously swap a pair of modes i and j whenever an interaction gate is applied between them. This permutation scheme can be implemented with no additional gate overhead by appropriately increasing the angle of the gate. Further, this approach leverages the efficient all-to-all connectivity of tweezer arrays. Even though the underlying interaction connectivity may be local, the spatial positions of two adjacent modes i and j are potentially distant after multiple swaps have occurred. This procedure also performs well for 1D interactions, due to a similar time-correlation effect; if two sites $x_{1}$ and $x_{2}$ interact at time step t, then $\sigma_{t}\left(x_{1}\right)=\sigma_{t+1}\left(x_{2}\right)$ and $\sigma_{t}\left(x_{2}\right)=\sigma_{t+1}\left(x_{1}\right)$. The effectiveness of this strategy can be seen in Fig. 6*A*, where it extends the available simulation time by two orders of magnitude.

For systems with higher-dimensional connectivity, the echo procedure should still improve the coherence of the system, as long as the permutation of sites results in an ergodic shuffling of atom positions. Assuming that the noise distribution is spatially random and independent, the average of accumulated phase differences should decrease as a square root of the number of time steps,

[19] $$\begin{array}{c} \frac{1}{t}\sum_{t^{\prime }\leq t}\left(h_{\sigma_{t^{\prime }}\left(j\right)}-h_{\sigma_{t^{\prime }}\left(i\right)}\right)∼\frac{1}{\sqrt{t}}, \end{array}$$

which results in the phase noise in Eq. **17** accumulating as $\sqrt{t}$. Without utilizing the specific structure of a given problem (as was the case in one dimension), it is unclear whether this scaling can be improved. Finding the optimal permutation strategy in a general setting is an important direction for future research. In Fig. 6*B*, we show the results of applying the echo procedure to a Hamiltonian defined on a 10 × 10 square lattice and observe an order-of-magnitude increase in the coherence time.

The results shown in Fig. 6 were obtained by simulating 50 atoms in 100 tweezers, interacting in a 1D-ring (Fig. 6*A*) and 2D-square-lattice (Fig. 6*B*) connectivity with periodic boundary conditions. The evolution under a nearest-neighbor tunneling Hamiltonian with the hopping rate J=1 and the time step τ=0.13, was implemented using free-fermion methods. The Hamiltonian evolution is split into parallel Floquet rounds and static, positional phase disorder following a Gaussian distribution with $\sigma_{\theta }=0.035$ is applied between each round.

## Data Availability

All study data are included in the main text.
